# The impact of individual and environmental interventions on income inequalities in sports participation: explorations with an agent-based model

**DOI:** 10.1186/s12966-018-0740-y

**Published:** 2018-11-01

**Authors:** David J. Blok, Frank J. van Lenthe, Sake J. de Vlas

**Affiliations:** Department of Public Health, Erasmus MC, University Medical Center Rotterdam, PO Box 2040, 3000 CA Rotterdam, The Netherlands

**Keywords:** Sports participation, Systems thinking, Agent-based modeling, Intervention, Income inequality

## Abstract

**Background:**

Income inequalities in sports participation are shaped by a system in which individuals and the environment interact. We developed an agent-based model (ABM) that could represent this system and used it to provide a proof-of-concept of its potential to explore the impact of individual and environmental interventions on reducing inequalities in sports participation.

**Methods:**

Our ABM simulates sports participation of individuals in the Dutch city of Eindhoven. In the model, sports participation is determined by an individual’s tendency to start sports (at a fitness center, sports club or self-organized), which is influenced by attributes of individuals (i.e. age, sex, income), sports facilities (i.e. price, accessibility) and the social environment (i.e. social cohesion, social influence). Sports facilities can adapt to changes in the demand by closures or startups, which in turn influence the tendency of individuals to participate in sport. We explored the impact of five interventions scenarios.

**Results:**

Explorative results show that providing health education, increasing the availability of sports facilities, lowering prices of facilities and improving safety levels can increase sports participation and modestly reduce absolute income inequalities in sports participation. The largest gain can be attained through health education, if the effect and reach is sufficiently large. Environmental interventions alone have a modest impact. Marked effects are only achieved after five to 10 years.

**Conclusions:**

ABMs have much potential to test the population-level effects of various interventions in the context of a system. Our study highlights the challenges of ABM development and reveals gaps in empirical data. With further refinements, our model could aid in understanding and finding optimal pathways to reduce income inequalities in sports participation.

**Electronic supplementary material:**

The online version of this article (10.1186/s12966-018-0740-y) contains supplementary material, which is available to authorized users.

## Background

Physical activity, including sports participation, prevents obesity, diabetes, cardiovascular diseases, and several cancers [1, 2]. Consistent evidence shows that adults with a lower socioeconomic position (SEP) are less likely to participate in physical activity and sports than their counterparts with a higher SEP [3, 4]. The reduction of socioeconomic inequalities in health behaviors through the promotion of sport among those in lower socioeconomic groups is a major challenge in public health.

Socioeconomic inequalities in sports participation are shaped by individual and environmental factors [5–7]. Individual cognitive factors, such as the intention of sports participation, derived from theories of behavioral change are important determinants of sports participation and are known to vary by age, sex and SEP [8]. However, the larger physical and social context in which behaviors are shaped and sustained also play an important role, as proposed in the social ecological model [9, 10]. Environmental characteristics of the built environment, such as the proximity to sports facilities, (the perception of) social safety, and economic factors, such as and price levels of sports facilities, contribute to the explanation of socioeconomic inequalities in sports participation [8, 11, 12]. Lower socioeconomic groups may reside more often in neighborhoods that are less supportive for certain health behaviors, including poorer access to facilities, and less favorable social circumstances [13, 14]. Lower socioeconomic groups are more likely to have a small social network and low social cohesion, which are relevant determinants of sports participations [11, 12, 15].

It is increasingly recognized that many of these determinants interact with and feedback on each other, creating a complex causal web [5, 7]. For example, people are to some extent sorted in neighborhoods based on characteristics, such as age, income, causing spatial clustering [16]. Characteristics of the individual and the residential neighborhood influence sports participation behaviors of individuals. As a feedback to individual behavior, the availability of sports facilities in neighborhood may change, which subsequently influences sports participation behavior, thereby changing social influences (or norms) that in turn may affect sports participation. At the same time, environments may reinforce individual cognitive factors: for example, stronger intentions to sports are associated with higher availability of sports facilities [17]. These complexities illustrate that sports participation arises from a system with multiple levels that are interconnected and interact. To be able to identify optimal ways to promote sports participation and to decrease inequalities in sports participation, there is a need to account for this complex non-linear system.

System approaches can capture a dynamic system in which individuals interact with each other and their environment [18]. Agent-based modeling is in particular promising, because it is the only tool that can simulate the dynamic processes of behavior change (here: in sports participation) at the population level by accounting for interactions between heterogeneous agents and their environment [5, 18, 19]. Furthermore, this approach is very suitable to test long term effects of intervention scenarios and compare them to a counterfactual [5, 20]. An agent-based model (ABM) contains autonomous agents (here: individuals and sports facilities) with specific characteristics (e.g. income level) and behaviors (e.g. sports participation) that can be followed over time. Behavioral rules describe how individuals interact with each other and the environment. An ABM captures feedback loops and adaptations of agents, e.g. a behavioral change of agents based on changing environments (such as more sports facilities) [21]. Recent ABM studies in social epidemiology have focused on dietary behaviors [22–24], social networks and obesity [25, 26], and daily walking [27, 28]. Thus far, no ABM has been developed to model sports participation.

In this paper we present a new ABM within the *Health Behaviors Simulation* (HEBSIM) suite [24]. Our aim is to represent a system that simulates sports participation among adults with different income levels to study income inequalities in sports participation, as emerging from interactions between individuals and sports facilities in neighborhoods of a city. Furthermore, we use our model to provide a proof-of-concept of its potential to explore the long term impact of interventions to promote sports participation and to assess how these interventions would affect income inequalities in sports participation.

## Methods

### General modeling approach

**Fig. 1:**
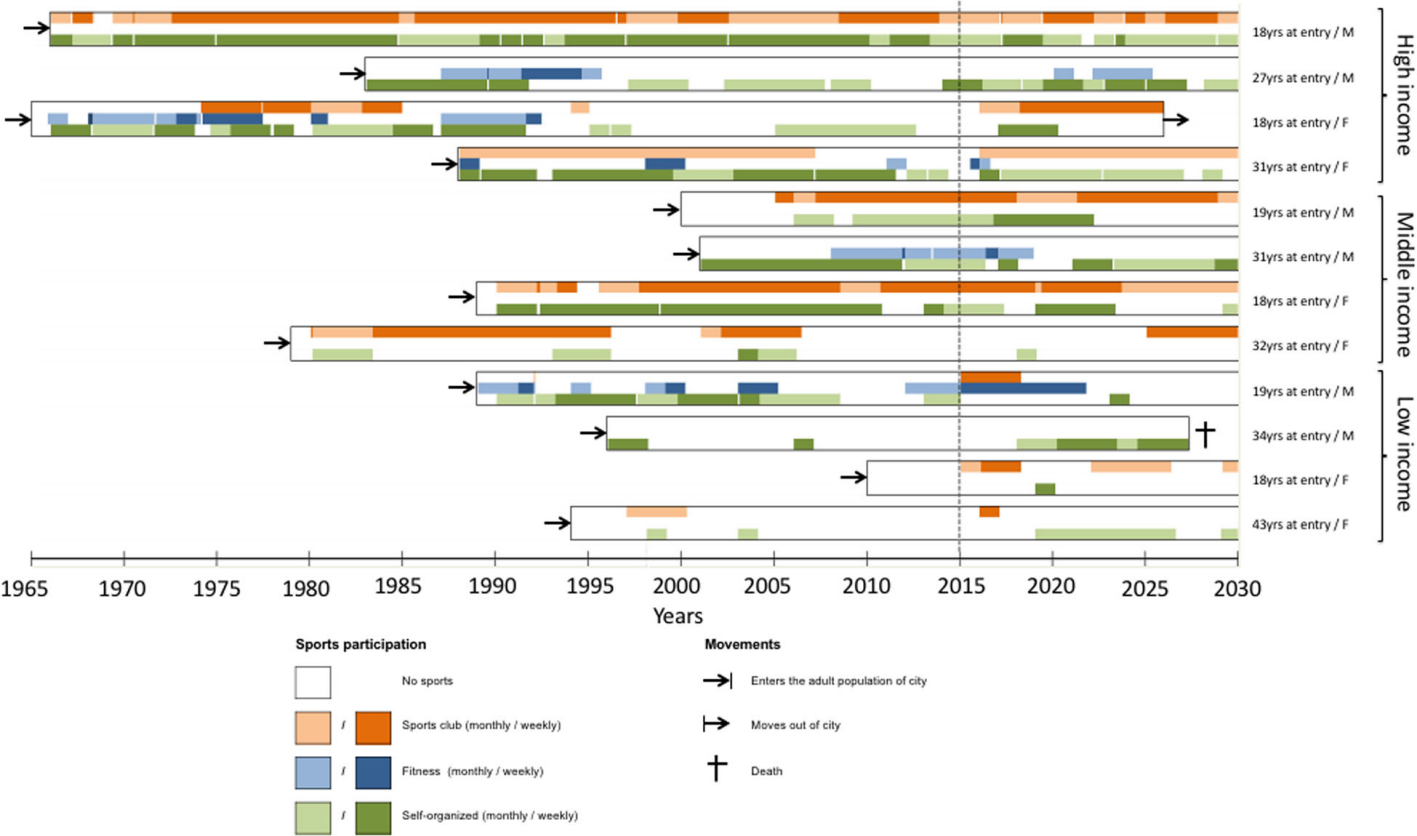
Example of twelve individuals as they may exist in the model in a selected period. Each bar represents the life of one individual, who can decide to start sports, quit sports or change the frequency of sports in the category fitness, sports club or self-organized. Individuals are grouped by income-level, i.e. high, middle and low-income. As individuals get older, they can die or move out of the city. Such individuals are replaced by new individuals to keep the population constant. The vertical dotted line represents the moment of the start of four interventions: providing health education, lowering prices of sports facilities, increasing availability of sports facilities and improving neighborhood safety

We modeled the city of Eindhoven in the Netherlands with its 88 residential neighborhoods using GIS data obtained from Statistics Netherlands, which was converted into a grid space with each cell of size 10 m X 10 m [24]. Individuals and sports facilities were modelled as agents. During the simulation, individuals become older, can die, and move out and into the city, based on patterns observed in empirical data [29, 30]. A time step in the model represents 1 month. Individuals entering the simulation do not participate in sport. During their life course, all individuals can start, quit and restart sports participation in three categories of sports as illustrated in Fig. 1: fitness, sports club (e.g. football, tennis), and self-organized (e.g. running) [31]. Whether, when and how often (i.e. monthly or weekly) an individual engages in sports participation is determined by the tendency to start sports, which is an index score of sports participation that represents how likely an individual would start sports. In our dynamic model, the tendency is used to determine the time until an individual starts sports participation. An individual with a high tendency to start sports is very likely to have a short duration until starting sports, while an individual with a very low tendency will likely have a very long duration, which may lead to never starting sports in his/her lifetime. We followed a social ecological approach in which the tendency to start sports results from interactions with attributes of individuals (i.e. age, sex, income), sports facilities (i.e. price, accessibility), and the (social) environment (i.e. safety, social cohesion, social influence) [9, 10]. This tendency may change over time as older age groups may less likely start sports due to for example decreasing ability or attitude [32]. Also, females and lower income groups may have lower tendency to start sports [32]. Expensive and remote sports facilities, lower neighborhood safety levels and lower social cohesion levels are modeled as barriers of sports participation [8, 11, 12, 17, 33]. Furthermore, the tendency to start sports is influenced by sports participation of direct neighbors (i.e. social norms) [15]. In response to sports participation behaviors of individuals, sports facilities can open or close over time, to which in turn individuals change sports participation behaviors. The text below provides an overview of the agent’s attributes, sports participation and behaviors of sports facilities. A detailed model description can be found in the Additional file 1.

### Attributes

The modeled population consists of 173,567 individuals between the age of 18 and 85 years, which were distributed according to the observed number per neighborhood in Eindhoven in 2014 [29]. Each individual is characterized by age, sex, income level and a tendency to start sports (index score). Age, sex, and income were assigned to each individual based on the distribution per neighborhood: the average age of the total population was 46 years, 49% was female, and 41%, 40% and 19% of the population had a low, middle, and high income, respectively [29]. The tendency to start sports is an individual characteristic that changes during the simulation. At creation, each individual is assigned an initial tendency to start sports participation. To account for heterogeneity in tendency between individuals, the initial tendency was drawn from a Gamma distribution with a mean of one (See Additional file 1: Section 2).

Sports facilities consist of fitness centers and sports club facilities, and can only be placed at a designated location for fitness centers and sports club facilities. These locations were assigned on the grid based on the actual number per neighborhood: i.e. 305 fitness center locations and 98 sports club locations in total. At the start of the simulation, fitness centers and sports club facilities are created based on the actual number of existing sports facilities per neighborhood in 2016: 30 fitness centers and 158 sport club facilities [34, 35]. The price level of a facility (dichotomized as ‘cheap’ or ‘expensive’) was assigned based on the fraction of expensive facilities (0.37 for fitness centers and 0.40 for sports club facilities). This fraction was determined by either the average monthly contribution-fee of a sports facility (cut-off: €20/month) or by the nature of the facility (See Additional file 1: Section 3) [36].

### Modeling sports participation

For each category of sports, the initial tendency was multiplied by an age group, sex, income, price, accessibility, safety, social cohesion and social influence score (See Additional file 1: Section 5). Age group scores of the young (18-35 yrs), middle (35-55 yrs) and old (55-85 yrs) age group, sex scores of males and females, and income scores of high-, middle- and low-income group were determined by calibrating the model against observed sports participation by age group, sex, and income level.

The price and accessibility scores depend on the selected fitness center or sports club facility. We assumed that sports participation in the category self-organized is not influenced by the price and accessibility score. To choose which sports facility to go to, individuals rank all fitness centers or sports club facilities based on a preference score and select the best fitness center or sports club facility (See Additional file 1: Section 5.1). The price score is set to 1.0, if the price level of the facility is cheap, and 0.85 if it is expensive. The latter is estimated using data from the GLOBE study [37]. The accessibility score is measured as *e*^−*β* ∙ *d*^, where *d* is the distance to a sports facility, and *β* is the distance decay. The distance decay of sports participation in the categories fitness and sports club were calibrated such that model outcomes match sports participation by categories of sports.

The perceived safety and social cohesion scores of an individual’s neighborhood were both derived from data [29]. Lastly, the tendency was assumed to increase with the social influence score, which is measured as the proportion of direct neighbors that engage in sports. Direct neighbors are defined as all those that live within a 50-m radius of the individual.

The time between entering the model and starting sports in a category of sports was modeled as exponential random variables. The rate of the exponential random variables is determined by the sports category-specific tendency multiplied by the mean frequency of sports in the city per year. The latter was calibrated against observed data on the overall sports participation in Eindhoven [34]. At the time of starting sports participation, the individual is categorized into either monthly or weekly sports participation (See Additional file 1: Section 5.4).

Quitting sports participation is determined by annual quitting probabilities. These were chosen at 0.28, 0.12 and 0.27 for the category fitness, sports club, and self-organized, respectively, based on national Dutch data [38]. Individuals who quit sports can restart sports after some time. The time between quitting sports and restarting sports is determined in a similar way to starting sports. In addition, individuals can also change the frequency of sports at the end of every year after starting sports. The probability of changing the frequency was derived from data about the intention to increase (i.e. from monthly to weekly) or decrease (i.e. from weekly to monthly) the frequency: 0.21 and 0.09, respectively [38]. An individual can also decrease or increase the frequency, whenever he/she starts multiple sports or quits sports when engaged in multiple sports. This occurs with an assumed probability of 0.5 (See Additional file 1: Sections 6 and 7).

### Modeling behaviors of sports facilities

During the simulation, new sports facilities can open and existing sports facilities can close in the city, and thus altering the composition of sports facilities. We assumed that on average one fitness center and one sports club facility close every year, and that on average also one fitness center and one sports club facility open in the city every year. In our model, the facility with the fewest members closes, upon which the location becomes vacant. A new facility is opened at a vacant location of a neighborhood with the highest demand for sports (See Additional file 1: Section 8).

### Model calibration and outcome

The calibration process was performed using a grid search in which eight unknown parameters were calibrated under three assumptions of variation in initial tendency (See Additional file 1: Section 9). The model was run for 50 years to make sure it reached equilibrium. Model outcomes in equilibrium were matched to the observed overall sports participation and sports participation by age group, sex, income and category of sports in Eindhoven [39]. Results in this paper are based on the best fitted model, i.e. assuming a Gamma (1.0, 0.5) for initial tendency (See Table 1, Additional file 1: Table S2 and Figure S4).

**Table 1:** Calibrated model parameters ^a^

Parameters	Value	(95% CI ^b^)
Frequency of sports per year, mean	153.5	(146.2–160.9)
Age group score		
18-35 yrs	1.0	–
35-54 yrs	0.157	(0.139–0.175)
> 55 yrs	0.148	(0.136–0.160)
Sex score		
Male	1.0	–
Female	0.659	(0.608–0.710)
Income score		
High income	1.0	–
Middle income	0.471	(0.428–0.514)
Low income	0.428	(0.387–0.470)
Distance delay score		
Fitness center	0.029	(0.028–0.031)
Sports club facility	0.027	(0.025–0.028)

^a^Assuming that initial tendency follows a Gamma(1.0,0.5)

^b^95% confidence interval

The outcome of interest is the annual overall sports participation and sports participation by income level. Absolute income inequality was calculated as the difference between sports participation in the high- and low-income group. Final model outcomes were the result of the average of 80 simulation runs. Intervals reflecting 95% uncertainty ranges were constructed by discarding the two highest and lowest outcome values.

### Interventions

To provide a proof-of-concept of the use of our model, we explored the impact of five intervention scenarios: 1) providing health education, 2) lowering prices of sports facilities, 3) increasing the availability of sports facilities, 4) improving neighborhood safety and 5) combining all previous interventions simultaneously (i.e. multilevel intervention) [10]. These intervention scenarios were chosen because they target both individual and environmental factors of the system. Also, the long term population-level impact of these interventions is generally unknown as they are difficult to test in real-life, requiring long follow-up studies. Results of intervention scenarios were compared to a scenario with no intervention for a period of 25 years, assuming everything remains unchanged. All interventions were assumed to be of immediate effect.

1. Health education is known to be have a positive effect on individual sports participation [40]. Here, we modeled health education as an increase in the tendency to start sports participation by a factor of 1.5. This factor was informed by the difference between the income score of high and low-income individuals (difference: factor of 2; See Table 1). The intervention was provided to 15% of the individuals who do not participate in sports. In separate scenarios, we further examined more optimistic scenarios in which the effect factor was gradually increased from 1.5 to 3 and the coverage from 15 to 50%.
2. Lowering prices of sports facilities was modeled by changing the price level of ‘expensive’ fitness centers and sports club facilities to ‘cheap’. As sports facilities are cheap from then on, it takes away the barrier of price level.
3. Increasing the availability of sports facilities in neighborhoods with low sports participation is hypothesized to increase sports participation, because it reduces distance [33]. In this scenario, one fitness center and one sports club facility were added to the five neighborhoods with the lowest sports participation. The newly created facilities are protected from closure in the next 10 years. In separate scenarios, we also explored the option of increasing the number of neighborhoods with new sports facilities.
4. Improving safety levels reduces a barrier to start sports participation [4]. In this scenario, the perceived neighborhood safety score was increased to the mean safety score of the entire city. This intervention only applies to neighborhoods with a safety score that is below the mean at the time of the start of the intervention. We also explored the impact of increasing the safety score to that of the neighborhood with the highest safety.

## Results

**Fig. 2:**
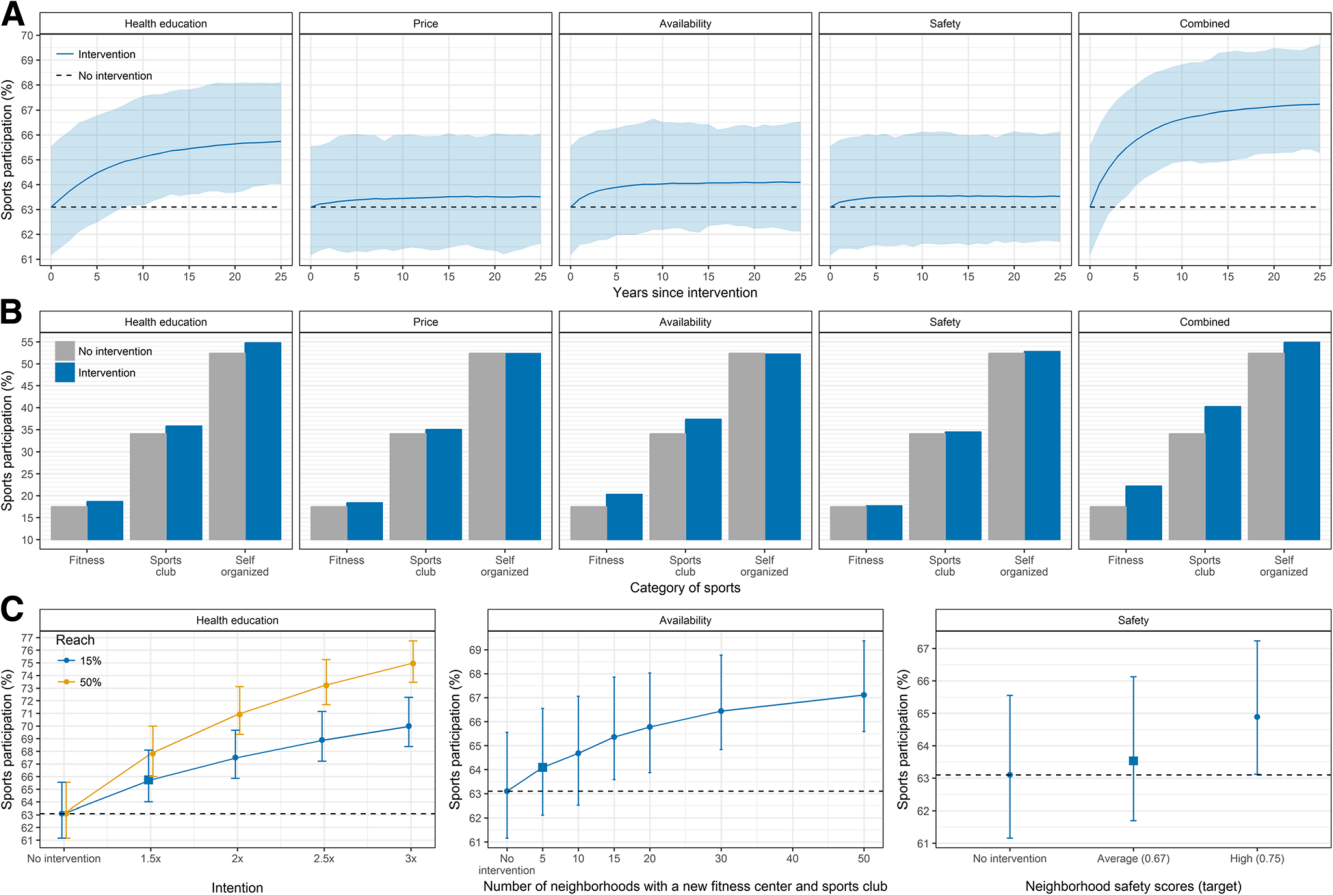
Predicted impact of five intervention scenarios on sports participation. **a** Impact on the proportion of total sports participation over time; **b** Impact on the proportion of sports participation by the category of sports after 25 years compared; **c** Impact of alternative intervention scenarios of health education, availability of sports facilities, and safety levels on sports participation after 25 years. Interventions scenarios include: 1) providing health education (effectiveness: 1.5x current tendency; reach: 15%), 2) lowering price level of expensive sports facilities to cheap; 3) increasing availability of sports facilities in five neighborhoods; 4) improving safety (target: average perceived safety score); 5) combining all previous interventions. The shaded area represents the 95% uncertainty interval due to parameter uncertainty and stochastic variation. Alternative intervention scenarios include: 1) varying effect of health education on an individual’s current tendency (1.5x, 2x, 2.5x, 3x current tendency) and its reach (15 and 50%); 2) increasing the number of neighborhoods with new sports facilities (10, 15, 20, 30, 50); 3) increasing the target level of safety (high level). The squares represent the outcomes of the intervention scenarios, and circles represent the outcomes of alternative intervention scenarios. The error bars represent the 95% interval due to parameter uncertainty and stochastic variation

Figure 2 presents the explorative impact of five intervention scenarios on sports participation. Providing health education, lowering the prices of sports facilities, increasing the availability of sports facilities and improving safety could increase sports participation from 63.1 to 65.7%, 63.5%, 64.1% and 63.5%, respectively (Fig. 2a). The large uncertainty around these predictions indicates that there is a chance that some scenarios may have no effect on sports participation at a population level, but the effect may also be two to eight times as large. Combined interventions could yield an increase of 4.1% points (from 63.1 to 67.2%) in sports participation, which equals an additional 7100 individuals starting sports. Interventions aimed at sports facilities only increase sports participation in the category fitness and sports club (Fig. 2b). The effects of interventions gradually increase over time with any marked effects usually only achieved after 5 to 10 years*.*

Figure 2c shows the potential impact if we could further increase the effect and reach of health education, the number of neighborhoods with new sports facilities and safety levels. Doubling the effect and reach of health education could increase sports participation to more than 70%. Building sports facilities in more neighborhoods could increase sports participation up to 67.1%. However, the gain of building facilities in an additional neighborhood diminishes after 15 neighborhoods. Further increasing the perceived safety score to 0.75 in all neighborhoods could increase sports participation to 64.9%.

**Fig. 3:**
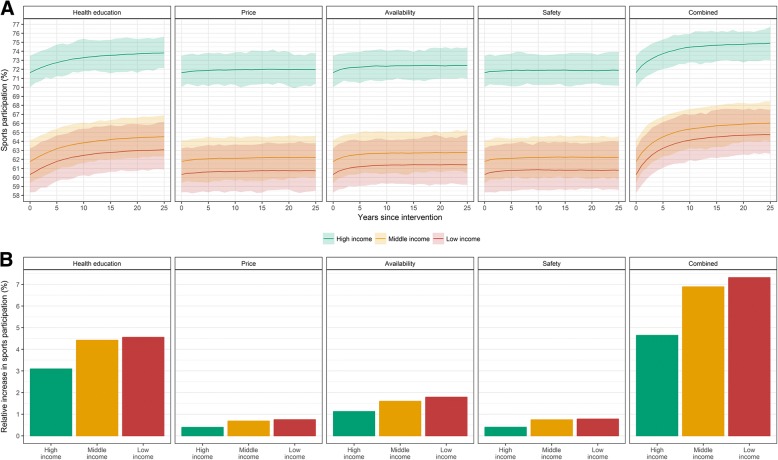
Predicted impact of interventions on sports participation rates by income group. **a** Impact on the proportion of total sports participation by income group over time; **b** Relative increase in sports participation after 25 years by income group; Interventions scenarios include: 1) providing health education (effectiveness: 1.5x current tendency; reach: 15%), 2) lowering price level of expensive sports facilities to cheap; 3) increasing availability in five neighborhoods; 4) improving safety (target: average perceived safety score); 5) combining all previous interventions. The shaded area represents the 95% uncertainty interval, which reflects parameter uncertainty and stochastic variation

Figure 3 shows the impact on income inequalities in sports participation. At baseline the modeled sports participation is 71.6%, 61.8% and 60.3% for the high-, middle- and low-income group, respectively. All interventions show a larger increase in lower income groups compared to the high-income group, indicating a decrease in absolute income inequalities (Fig. 3b). Again, combining all interventions yields the largest impact: absolute income inequalities in sports participation between the high- and low-income groups drop from 11.3 to 10.1% after 25 years.

**Fig. 4:**
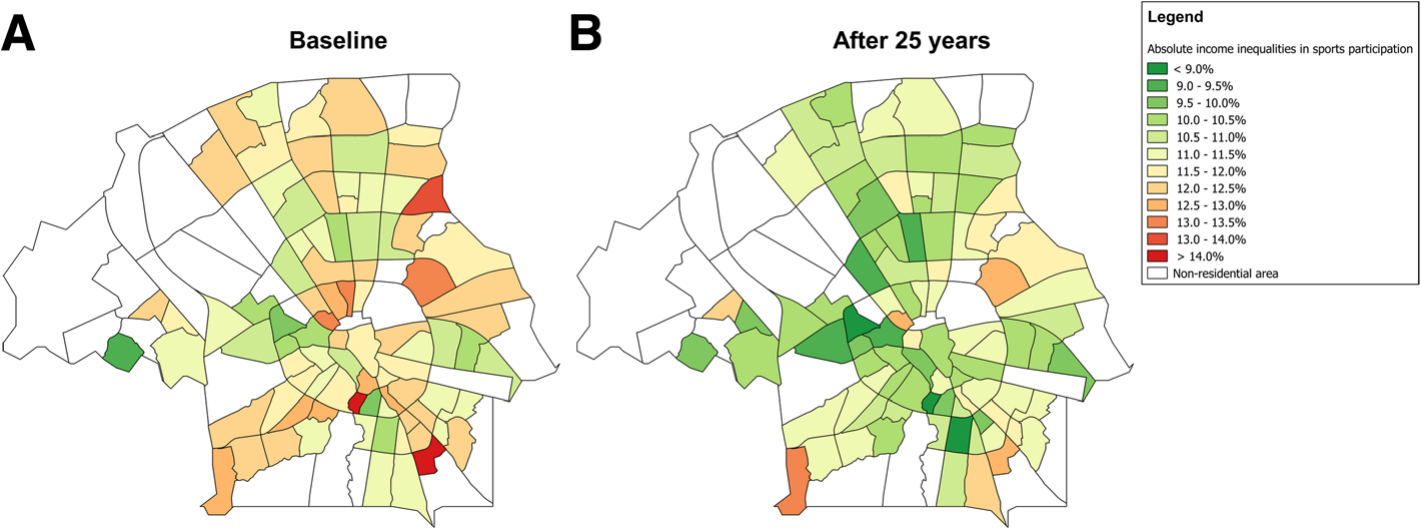
Predicted impact of interventions on absolute income inequalities in sports participation rates. **a** Absolute income inequalities of sports participation at baseline; **b** absolute income inequalities of sports participation after 25 years with combined interventions. Absolute income inequalities are the difference between the proportion of sports participation in the high- and low-income group

Absolute income inequalities in sports participation within neighborhoods are predicted to decrease in almost all of the neighborhoods (See Fig. 4). However, neighborhoods with large inequalities at baseline will continue to have the largest inequalities after the interventions. These neighborhoods are primarily situated at the periphery of the city.

## Discussion

This study presents an ABM for income inequalities in sports participation. It provides a first example of how the concept of complex systems-thinking in sports participation behavior could be translated into an ABM. The need to embrace this concept is now increasingly being recognized [41–43]. Furthermore, we used our model to illustrate its potential to explore the long term population-level effects of various interventions in the context of a system. Although our explorative results of interventions are secondary to the proof-of-concept, these may still provide some useful insights.

Explorative results show that providing health education, increasing the availability of sports facilities, lowering prices of facilities and improving safety levels can increase sports participation. Also, they modestly reduce absolute income inequalities in sports participation. Our model suggests that the largest gain can be attained through health education, if the effect and reach is sufficiently large. Interventions targeting prices of sports facilities, the availability of sports facilities and safety levels solely generally have a modest impact. Combined interventions yield the highest impact, suggesting that a multilevel approach is favorable over single interventions. This result is in line with the key premises of social ecological models [10]. However, the impact of combined interventions shows overlap, represented by the fact that the sum of the impact of single interventions is greater than that of combined interventions. This implies that adding another intervention does not necessarily result in a significant additional impact, because they affect the same people. This is also a reason why we need ABMs; such effects are difficult to measure in real-life due to the complexities to design and conduct controlled experiments. ABMs can aid in testing the added value of multilevel interventions [10, 44].

Significant population-level effects are usually obtained after 5 or 10 years, which supports the general idea that policies and interventions may affect population health slowly [45]. It should be noted that we even assumed the interventions to have an immediate impact, so that real impact likely appears after an even longer time. As in most cases direct evidence for the long-term population-level effect of an intervention is unknown, ABMs can be useful for long-term planning of intervention studies.

Although we managed to model a system of sports participation and to replicate sports participation rates of the city of Eindhoven, it remains a simplified representation of the processes driving sports participation and there is substantial room for further improvements, especially if more (detailed) data become available. Thus, our explorative findings of the interventions cannot be interpreted for policy-purposes yet, but are indicative at most. Our model could be regarded as a starting point or example for future models to be developed in this area.

Developing an ABM goes hand in hand with challenges, including design, identifying interactions, and data and parametrizing models. The design of an ABM is subjected to the purpose of the model. Generally, it is advised to start simple, because such models can reveal new theoretical ideas and knowledge gaps [46]. As the aim of this study was to provide a proof-of-concept of the usage of ABM, we started off by modeling sports participation based on a simple social ecological model, including interactions between individual and environmental factors. As a result, we disregarded typical behavioral change theories, such as Theory of Planned Behavior [47]. Individual cognitive factors have been simplified and captured by the model-specific concept called “tendency”. (Unmeasured) variation in tendency between individuals is to a large extent expressed through the stochastic nature by which initial tendency has been assigned and used. The process of personal decisions making is one area that would be first on the list to be refined. This could be done by modeling behavioral change theories. However, this is only recommended, if the purpose of the model is to evaluate possible changes in the attributes of these theories, such as attitude or intention.

The identification of relevant factors underlying sports participation at the individual and population level were based on: (1) a review of the literature about their importance to explain sports, (2) their relevance for potential intervention targets. Obviously, not all relevant factors will have been included, making the scope of the model purposely narrow. The paradox is that if a model has a large number of factors (i.e. more complicated), it will be more difficult to interpret and validate [48]. It is therefore advised to start by fully understanding the dynamic processes within a relatively simple model, before embarking upon more complex, comprehensive models [46].

There are a number of simplifications in our model. First, starting sport, changing frequency and quitting sports are modelled completely independently. For example, quitting sports was based on rates and did not depend on an individual’s tendency. To be able to model these processes more accurately, data are needed that follow individuals’ sports participation behavior in different categories of sports over time and include information about key drivers of starting, quitting and changing frequency of sports participation. Second, dichotomization of price levels, which may to some extent explain why the impact of lowering prices of sports facilities on sports participation turned out to be very modest. A continuous price level might have been a better approach. However, this would require data about the relationship between price levels and sports participation. Third, the social environment was restricted to direct neighbors only. Close contacts, such as friends, are not considered in this model, even though this has been argued to be most influential with regard to health behaviors [45]. In order to model close contacts, network data is required which also include sports participation behaviors of the respondents. Fourth, our model did not account for effect of sports participation started in childhood. It is known that sports participation in adulthood is partly determined by the sports participation during childhood or adolescence. Moreover, these decisions in childhood are largely influenced by the parents’ SEP, which partly explains income inequalities at later stages of life. Further extending the model by incorporating a life course perspective might therefore be an important next step [49, 50].

ABMs as any kind of mathematical models require thorough evaluation, which includes verification, calibration and validation. To assure some realism in our models, we based parameter values and decision rules directly on empirical data and studies where possible. Validation of the projected trend and impact of intervention scenarios with fully independent data would increase reliability, but was unfortunately not possible due to lack of such data, which is a common problem in agent-based modeling in general. This is a crucial step if we want these models to become an aid for policy.

Modeling findings should always be interpreted in the context of the assumptions made about the intervention scenarios. In this study, explorations of the impact of interventions were primarily intended to illustrate the potential of ABM; therefore, we used rather extreme (unrealistic) assumptions. For example, we assumed that all interventions have an immediate effect, while in reality this likely is a much slower process which may even take years (e.g. building new facilities). Our predicted time frame for interventions to reach large impacts may therefore be underestimated. Also, we assumed that the composition of sports facilities is optimal at baseline. Our model was run for 50 years to reach equilibrium allowing the locations of sports facilities to be optimized through closures and startups. The impact of interventions targeting sports facilities may have been slightly larger if we would have started from a situation in which the composition of sports facilities is suboptimal. Furthermore, long term projections of interventions should always be interpreted with care, as populations may change over time in terms of size, age structure and income distribution per neighborhood.

## Conclusion

We conclude that ABMs have potential for developing and testing the population-level effects of various interventions in the context of a system. Our study illustrates the level of complexity of an ABM and highlights gaps in empirical data. Explorative findings highlight that increasing sports participation and reducing income inequalities in sports participation requires sustained effort with population-level effects only being visible in the long-term. With further refinements, ABMs may eventually become useful tools to support decision-makers in answering questions in public health arising from complex interactions.

## Additional file

Additional file 1: Supplemental material: Model description. (PDF 696 kb)

## Abbreviations

ABM: Agent-based model
SEP: socioeconomic position
