# Supplementary material for: The impact of individual and environmental interventions on income inequalities in sports participation: explorations with an agent-based model
Source: Int J Behav Nutr Phys Act. 2018 Nov 1;15:107. doi: 10.1186/s12966-018-0740-y (PMC6211418; doi:10.1186/s12966-018-0740-y)
Supplement: Supplementary file 1 — Supplemental material: Model description. (PDF 696 kb) [file 12966_2018_740_MOESM1_ESM.pdf]

Model description

David J. Blok[1], Frank J. van Lenthe[1], Sake J. de Vlas[1]

[1]Department of Public Health, Erasmus MC, University Medical Center Rotterdam, The Netherlands

This document contains supplementary material to accompany the paper *'The impact of individual and environmental interventions on income inequalities in sports participation: explorations with an agent-based model'*. It provides a full description of the HEBSIM-sports model and its calibration.

Contents

S1 General approach 2

S2 Attributes of individuals 4

S3 Attributes of sports facilities 4

S4 Demographic processes 4

S5 Starting sports participation 5

S5.1 Selection of a sports facility 6

S5.2 Tendency 7

S5.3 Time of starting sports 9

S5.4 Frequency of sports participation 10

S6 Quitting sports participation 10

S7 Changing frequency of sports participation 11

S8 Sports facility closures and startups 11

S9 Model calibration 12

References 15

S1 General approach

Individuals and sports facilities were modelled as agents that interact with each other within neighborhoods of a city. We simulated the city of Eindhoven, situated in the South-Eastern part of the Netherlands. A grid was constructed based on a GIS map of Eindhoven, obtained from Statistics Netherlands [1]. The size of the city is 88km^2 with 116 neighborhoods of which 88 are residential neighborhoods. The map was rasterized such that each grid cell represents 10 m x 10m in size. Each grid cell can be occupied by an individual and/or a sports facility.

The simulated population includes individuals between the age of 18 and 85 years. The population of Eindhoven consist of 173,567 persons of age 18-85 years in 2014, living across 88 residential neighborhoods [2]. The numbers per neighborhood range from 138 to 4,965 (see figure S1A). The actual number of individuals was randomly placed in a neighborhood. Each individual is characterized by attributes including age, sex, income, tendency to start sports and location.

The model considers two types of sports facilities: fitness centers and sports club facilities (e.g. football, tennis). Fitness centers and sports club facilities can only be placed at a designated location for fitness centers and sports club facilities, respectively. These locations are assigned on the grid based on the actual number per neighborhood: i.e. 305 fitness center locations and 98 sports club locations in total (see figure S1B and S1C) [2, 3]. At the start of the simulation fitness centers and sports club facilities are created based on the actual number of existing sports facilities per neighborhood: 30 fitness centers and 158 sports club facilities. These were identified by accessing the national fitness register and sports club database of Eindhoven in February 2016. Only one fitness center is allowed to be placed at one designated fitness center location, but multiple sports clubs are allowed to be placed at one designated sports club location, as is the case in the city of Eindhoven. Sports facilities are characterized by price level and location.

In the model, individuals get older, die, or move out of the city, and they can engage in sports participation. During their life course, these individuals can start, quit and restart sports participation in three categories of sports: fitness, sports club (e.g. football, tennis), and self-organized (e.g. running) [4]. Whether, when and how often (i.e. monthly or weekly) an individual engages in sports participation is determined by the tendency to start sports, which is an index score that represents how likely an individual would start sports. The tendency is used to determine the time until an individual starts sports participation. In response to sports participation behaviors of individuals, sports facilities can open or close over time, to which in turn individuals may change their sports participation behaviors.

(A) Individuals

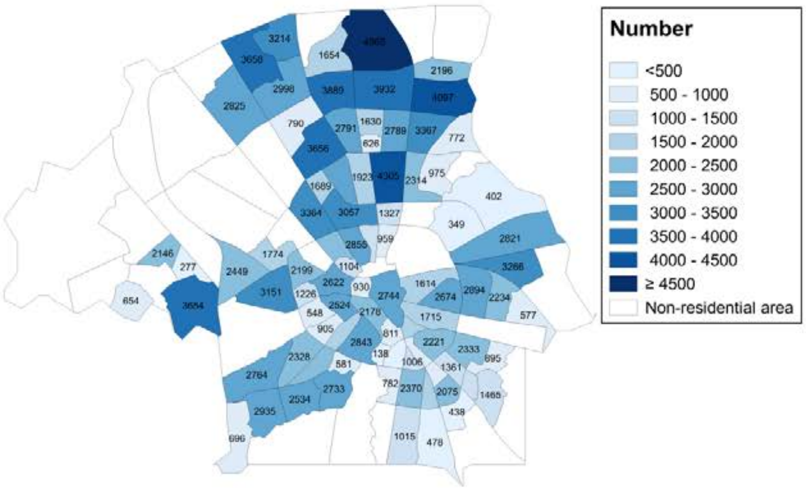

(B) Fitness centers

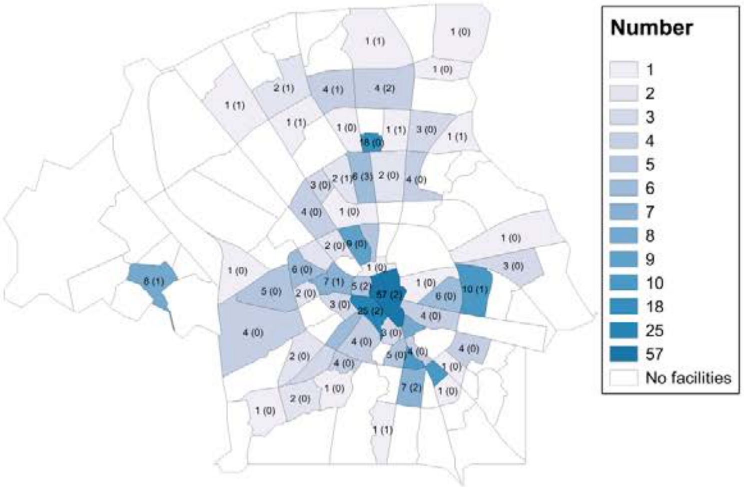

(C) Sports clubs

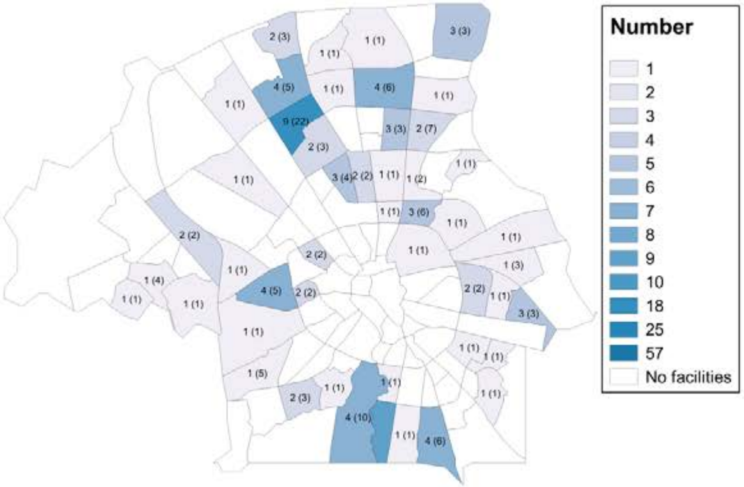

Figure S1. Map of Eindhoven with (A) the number of individuals between the age of 18-85 years, (B) the number of fitness center locations, (C) the number of sports club locations per neighborhood. The map contains in total 116 neighborhoods. Blue neighborhoods in (A) represent residential neighborhoods (in total 88). The blue areas in (B) and (C) represent neighborhoods with fitness center locations and sports club locations, respectively. The numbers between brackets represent the number of fitness centers and sports club facilities at the start of the simulation. Facilities can only be located in neighborhoods with known locations. Darker areas represent higher numbers per neighborhood. Source: Municipality of Eindhoven [2], national fitness register [5] and sports club database of Eindhoven [3]

S2 Attributes of individuals

Each individual is characterized by individual-level attributes, including age, sex, income level and a tendency to start sports. Age, sex, and income were assigned to each individual based on the observed distribution per neighborhood (See table S1) [1, 2].

Table S1. Characteristics of the modelled population

Parameter	City level	Neighborhood level (Range [a]: min – max)	Source
Age (mean 46 years)			[2]
18-35yrs	33%	4 – 64%	
35-55yrs	35%	20 – 61%	
55-85yrs	32%	7 – 67%	
Sex – Female	49%	39 – 57%	[2]
Income			[1]
Low	41%	16 – 54%	
Modal	40%	12 – 50%	
High	19%	5 – 55%	

[a] Range between 88 neighborhoods

The tendency to start sports is an individual characteristic that changes during the simulation. At creation, each individual is assigned an initial tendency to start sports participation (index score with a mean of 1.0). The initial tendency to start sports of individual i was randomly assigned following a Gamma distribution with mean 1.0 and shape equal to k, i.e. Gamma(1.0, k). Lower values of k represent more individual variation. We calibrated the model under three assumptions of the shape parameter k: 0.5, 1.0, 3.0. See S9 for more information about calibration.

S3 Attributes of sports facilities

The price level, either 'cheap' or 'expensive', of a sports facility was assigned based on the fraction of expensive fitness centers and sports club facilities. For fitness centers, this fraction was determined by the average monthly contribution-fee of a fitness center. All fitness centers with a price-level above €20 per month were considered expensive [6]. The fraction of expensive sports club facilities was determined by the type of sports: golf, tennis and equestrian sports were considered relatively expensive, while football, swimming, athletics were considered relatively cheap. The fractions of expensive fitness centers and sports club facilities were 0.37 and 0.40, respectively

S4 Demographic processes

During the simulation, individuals become older, and can die, move out of the city and new individuals can move into the city. The age of death of an individual was determined upon entrance

to the city based on the Dutch survival curve of 2014 (See figure S2) [1]. At the age of death, the individual is removed from the simulation. Also, all individuals above the age of 85 years are removed from the modelled population, as the model only includes individuals between the age of 18 and 85 years.

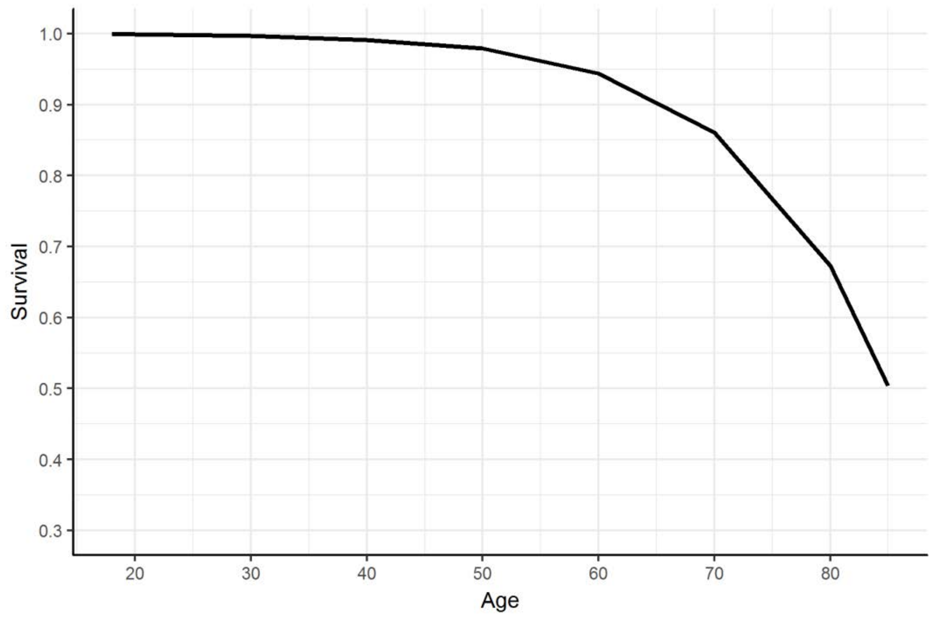

Figure S2. Survival rate of people at the age of 18 years. Source: Statistics Netherlands (CBS) [1]

Every year a fraction of the individuals migrates to another city. This fraction was derived from the observed annual numbers of out-migration per neighborhood to other cities. Around 7.5% of the population migrates to another city per year (range between neighborhoods: 2 – 18%) [2].

To keep the population per neighborhood at a constant size throughout the simulation runs, individuals that are removed (either due to death, old age or migration) are replaced by new individuals. The age and income level of the new individual adheres to the observed age, sex and income distribution of the neighborhood. This assures that also the neighborhood age, sex and income distribution remain unchanged.

S5 Starting sports participation

In our model, individuals can start sports in one or more categories c:

1) Fitness
2) Sports club (e.g. football, tennis)
3) Self-organized (e.g. running).

As a result, each individual has three tendencies: tendency to start sports in the category (1) fitness, (2) sports club and (3) self-organized. These tendencies are influenced by attributes of individuals (i.e. age, sex, income), and the (social) environment (i.e. safety, social cohesion, social influence). Additionally, the tendency to start sports in the category fitness and sports club depend on the characteristics of fitness centers and sports club facilities (i.e. price level, accessibility), respectively.

Therefore, to calculate the tendency to start sports in the category fitness and sports club, each individual selects a fitness center and sports club facility first (See S5.1).

To determine whether and when an individual starts sports, a duration until starting sports participation is drawn following an exponential distribution based on an individual's tendency for each of the three categories of sports separately (See S5.2 and S5.3). Each tendency of an individual is in this way translated into a waiting time until starting sports. As the duration until sports participation is drawn for each of the three categories of sports separately, an individual can engage in sports in one or more categories of sports simultaneously. The duration until starting sports for each category of sports c is determined in the following situations:

1) At the time of entering the simulation. An individual enters the simulation either at the start of the model or whenever he/she migrates into the city. Each individual starts without engaging in any sports participation. A duration until starting sports is then determined for each of the three categories of sports separately.
2) At the time an individual quits sports participation in the category of sports c. Upon quitting sports, a duration until restarting sports of that same category c is calculated.
3) At the time the fitness center or sports club facility, of which the individual is a member, closes. A closure forces an individual to quit sports in the category of sports to which the facility belongs to. Upon quitting sports, a new duration until restarting sports in that same category of sports is calculated.
4) At the time a new fitness center or sports club enters the simulation and the individual is not engaged in sports in the category fitness or sports club yet. The individual determines whether the new fitness center or sports club facility is preferred over the currently selected fitness center or sports club facility. If that is the case, a new duration until starting sports is drawn.

S5.1 Selection of a sports facility

A preference score is assigned to all fitness centers or sports club facilities in the city using equation (1). The fitness center or sports club facility with the highest preference score is selected.

$$preference_{i,f,c} = \frac{1}{d(l_i, l_{f,c})} \cdot priceS(p_{f,c}) + \varepsilon_f \tag{1}$$

With:

$d(l_i, l_{f,c})$ is the Euclidian distance between the location l of individual i and the location l of sports facility f of category of sports c (i.e. fitness or sports club).

$priceS(p_{f,c})$ is the price score, which is determined by the price level p of sports facility f of category of sports c. The price score was pre-set at 1.0, if the price level p is cheap. The price score of expensive sports facilities was derived from the GLOBE study (wave 2004). Respondents indicated whether an expensive facility was considered a barrier to start physical activity. In total, 15% of the respondents indicated this was case. Based on this result, we set the price score of expensive facilities to 0.85, which can be considered as a crude proxy.

ε_f is a random variable following a Normal distribution with μ= 0 and σ = 0.05. This 'random noise' was added to represent bounded rationality. Individuals do not always choose a sports facility with perfect rationality [7].

S5.2 Tendency

The tendency to start sports of individual i at time t is calculated for each of the three categories of sports c separately. If the category of sports c is fitness or sports club, it is calculated as:

$$tendency(t)_{i,c,f} = initial\_tendency_i \cdot a\_groupS(a(t)_i) \cdot sexS(sx_i) \cdot incomeS(il_i) \cdot accessS(l_i, l_{f,c}, \beta_c) \cdot priceS(p_{f,c}) \cdot safetyS(n_i) \cdot s\_cohesionS(n_i) \cdot s\_influenceS(t)_i \quad (2A)$$

The tendency to start sports in the category self-organized does not depend on characteristics of a sports facility, and is therefore calculated as:

$$tendency(t)_{i,c} = initial\_tendency_i \cdot a\_groupS(a(t)_i) \cdot sexS(sx_i) \cdot incomeS(il_i) \cdot safetyS(n_i) \cdot s\_cohesionS(n_i) \cdot s\_influenceS(t)_i \quad (2B)$$

With:

$intitial\_tendency_i$ is the initial tendency to start sports of individual i. It is an index score with a mean of 1.0. See S2 for more information about how initial tendency is assigned.

$a\_groupS(a(t)_i)$ is the age group score of an individual i with age a at time t. Age was categorized here into three groups: young (18-35yrs), middle (35-55yrs) and old (55-85yrs). The score of the age group young was pre-fixed at 1.0. The remaining scores were calibrated to match sports participation by age group.

$sexS(sx_i)$ is the sex score of an individual i with sex sx. The score of males was pre-fixed at 1.0. The score of females was calibrated to match sports participation by sex.

$incomeS(il_i)$ is the income score of an individual i with income level il. Income was categorized into three groups: high-, middle- and low-income. The score of the high-income group was pre-set at 1.0. The scores of the remaining income categories were calibrated to match sports participation by income level.

$accessS(l_i, l_{f,c}, \beta_c)$ is the accessibility score, which is measured as:

$$accessS(l_i, l_{f,c}, \beta_c) = e^{-\beta_c \cdot d(l_i, l_{f,c})} \quad (3)$$

With:

$d(l_i, l_{f,c})$ is the distance between the location l of individual i and the location l of sports facility f (i.e. fitness center, when c = fitness, or sports club facility, when c =sports club).

β_c is the distance decay of category of sports c (i.e. fitness or sports club). The distance decay of the category fitness and sports club were calibrated to match the observed proportion of people doing sports in the category fitness and sports club.

Accessibility was not considered for sports in the category self-organized (e.g. running), because it can be started from home.

$priceS(p_{f,c})$ is the price score, which is based on the price level p of the selected sports facility f (i.e. fitness center, when c = fitness, or sports club facility, when c =sports club). The price score was pre-set at 1.0, if the price level p is cheap. The price score of expensive sports facilities was set to 0.85, based on the GLOBE study (See S5.1). As the tendency to start sports in the category self-organized is not influenced by price score, we implicitly assume that the price level is 'cheap'.

$safetyS(n_i)$ is the safety score of the residential neighborhood n of individual i. This score is based on the perceived neighborhood safety as derived from the data from the Municipality of Eindhoven [2]. The perceived safety was assessed by a survey among residents of Eindhoven. The score ranges from 0.0 to 1.0, where 1.0 reflects perfect safety. Figure S3A shows the perceived safety score per neighborhood. The mean perceived safety score of Eindhoven is 0.67.

$s\_cohesionS(n_i)$ is the social cohesion score of the residential neighborhood n of individual i. This score is based on the perceived neighborhood social cohesion level as derived from the data from the Municipality of Eindhoven [2]. The perceived social cohesion score was assessed by a survey among residents of Eindhoven and included questions about social connections, trust, feeling at home [2]. The score ranges from 0.0 to 1.0, where 1.0 is perfect social cohesion. Figure S3B shows the social cohesion score per neighborhood.

$s\_influenceS(t)_i$ is the social influence score at time t. The tendency increases proportionally with the number of direct neighbors engaging in sports. The social influence score is measured as:

$$s\_influenceS(t)_i = 1 + \alpha\left(\frac{DN(t)_i}{N(t)_i}\right) \quad (4)$$

With:

α indicates the strength of social influence. In this study, we set α to 1.0, which implies that social influence can increase the tendency by a factor of two. This is reasonable as the difference in tendency between individuals with a high income is twice as high as those with a low income (See table 1 in manuscript).

$DN(t)_i$ is the total number of direct neighbors of individual i that engage in sports at time t. Direct neighbors are those that are living within a 50-meter radius of the individual.

$N(t)_i$ is the total number of direct neighbors of individual i at time t.

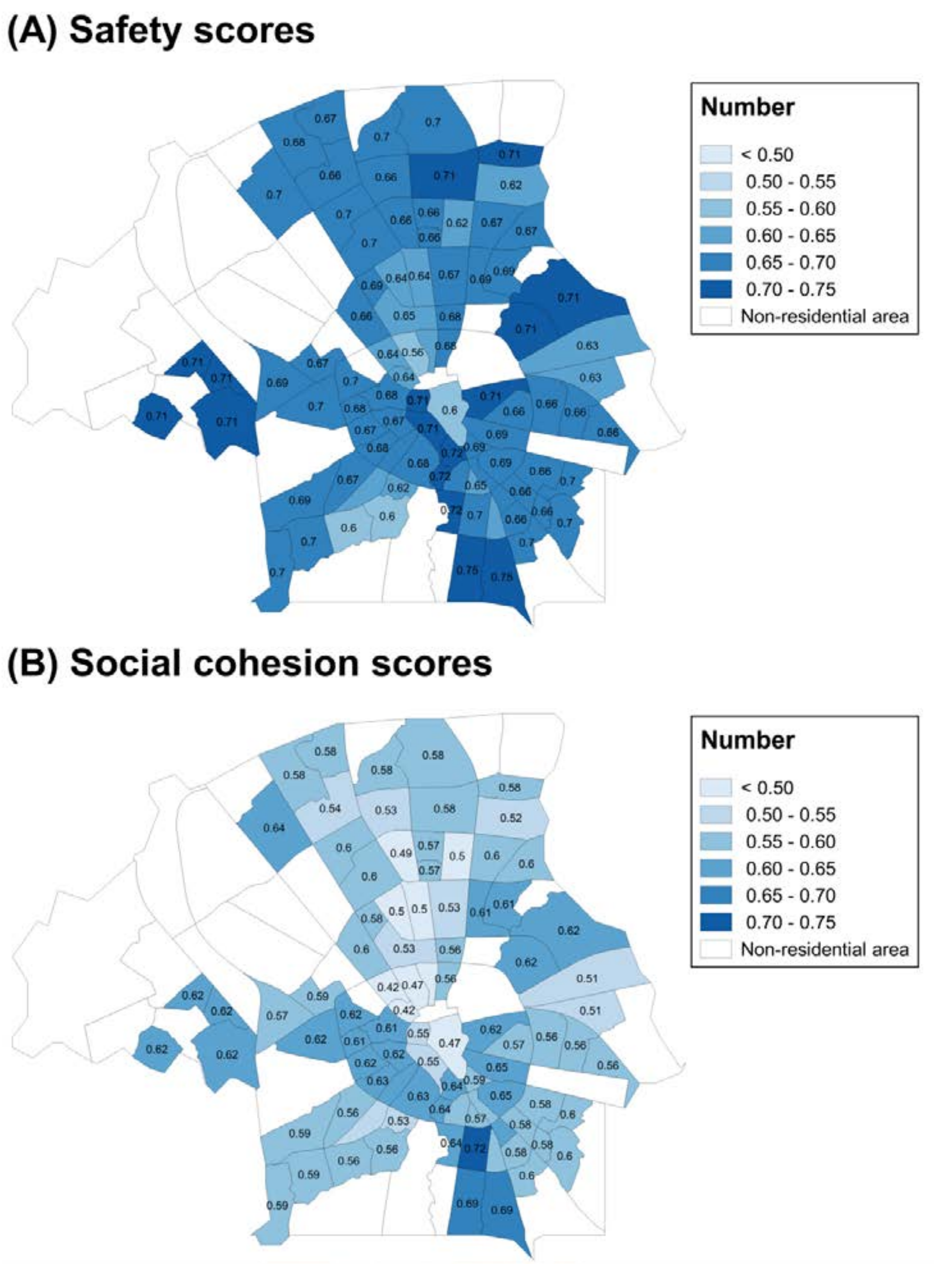

Figure S3. Perceived neighborhood A) safety scores and B) social cohesion scores. Source: Municipality of Eindhoven [2].

S5.3 Time of starting sports

The time of starting sports participation of individual i in category of sports c (i.e. fitness, sports club, self-organized) is determined as follows:

$$time\_sport_{i,c} = t + duration_{i,c} \tag{5}$$

With:

t is the current time

$duration_{i,c} \sim Exponential(\lambda_{i,c})$ is the duration in years until starting sports by individual i in category of sports c. The duration until starting sports follows an exponential distribution with rate equal to $\lambda_{i,c}$

The rate of the exponential random variables $\lambda_{i,c}$ is determined as:

$$\lambda_{i,c} \begin{cases} \frac{1}{nSports \cdot tendency(t)_{i,c,f}} & , if\ c = fitness\ or\ sports\ club \\ \frac{1}{nSports \cdot tendency(t)_{i,c}} & , if\ c = self\ organized \end{cases} \quad (6)$$

With:

$nSports$ is the mean frequency of sports per year in the city. This parameter was calibrated such that model outcomes match the observed sports participation in Eindhoven.

$tendency(t)_{i,c,f}$ & $tendency(t)_{i,c}$ is the tendency to start sports of individual i at time t of category c (See equation 2A & 2B).

S5.4 Frequency of sports participation

At the time of starting sports in a category of sports c, the frequency of sports participation is determined for that particular category of sports. The individual is categorized into either: (1) 'monthly sports participation', or (2) 'weekly sports participation'. The actual frequency of category c of an individual is determined by multiplying the individual's tendency to start sports of category c by the mean frequency of sports per year in the city. The individual is categorized into "monthly sports participation", if this number is between 12 and 40, and "weekly sports participation", if above 40. If this number is smaller than 12, we assumed that the individual does not participate in sports in that particular category of sports, and a new duration until starting sports in that particular category of sports is calculated.

S6 Quitting sports participation

Quitting sports can occur during the following events:

1) At the end of every year since the start of sports participation
2) At the time a sports facility closes (only for sports participation in the category fitness or sports club)
3) At the time of starting sports in another category of sports

At the end of every year since the start of sports participation, an individual quits sports participation with a probability of 0.28, 0.12, and 0.27 in the category fitness, sports club, and self-organized, respectively. These probabilities were based on a survey that included questions about the intention to quit sports in the next year [8]. A closure of a sports facility forces an individual to quit sports in either the category fitness or sports club. Upon quitting sports in a particular category of sports, a new duration until restarting sports for that particular category is calculated (See S5.3).

Starting sports in another category of sports can cause an individual to quit current sports, if the frequency of current sports is ‘monthly’. The reasoning behind this is that due to possible time constraints an individual may decide to quit sports he/she currently engages in. The probability of quitting current sports as a result of starting sports in another category was arbitrarily chosen to be 0.5.

S7 Changing frequency of sports participation

Changing frequency of sports participation (i.e. ‘monthly’ or ‘weekly’) can occur during the following events:

1) At the end of every year since the start of sports participation
2) At the time of starting sports in another category of sports
3) At the time of quitting sports while an individual still does sports in another category of sports

At the end of every year since the start of sports participation in any category of sports, an individual can increase frequency from ‘monthly’ to ‘weekly’ and decrease frequency from ‘weekly’ to ‘monthly’ with a probability of 0.21 and 0.08, respectively. These probabilities were derived from a Dutch survey asking respondents whether they would increase, decrease their current frequency of sports, or are happy with their current frequency of sports [8].

Starting sports at another category of sports can decrease the frequency of current sports from ‘weekly’ to ‘monthly’. The probability of decreasing the frequency of current sports when starting sports in another category of sports was arbitrarily chosen to be 0.5.

Quitting sports in a particular category of sports (either fitness, sports club or self-organized) while still engaging in sports of another category may trigger an increase in frequency of sports from ‘monthly’ to ‘weekly’. The probability of increasing frequency during this event was arbitrarily chosen to be 0.5.

S8 Sports facility closures and startups

In the model, sports facilities can be closed and new sports facilities can be opened in the city. The number of fitness center and sports club facility startups in the province of Noord-Brabant were 14 and 8, respectively, in 2013 [9]. As Eindhoven has approximately 10% of the population of Noord-Brabant, we crudely assumed that on average one fitness center and one sports club facility are opened every year. We also assumed that on average one fitness center and one sports club facility close every year [9]. This is reasonable as the composition of sports facility in Eindhoven has fairly stable been stable in recent years [9].

The time of closures and startups of a fitness center and sports club facility are drawn from an exponential distribution with a rate of 1.0 per year. After each startup or closure, the time of the next startup or closure is determined.

Closure: At the time of closure of a fitness center or sports club facility, the fitness center or sports club facility with the lowest number of members is closed. The location of the sports facility becomes vacant.

Startup: At the time of startup of a fitness center or sports club facility, a new fitness center or sports club facility is opened. The location of the new facility is determined by the demand for sports. We assume that sports facilities prefer establishment in neighborhoods where there is a large demand. The demand in neighborhoods with vacant facilities is assessed using equation (7). The neighborhood with the highest demand is selected. The new fitness center or sports club is then randomly located at one of the vacant locations in that neighborhood.

$$Demand_s = \frac{SP(t)_{c,n}}{1 + Fac(t)_{c,n}} \tag{7}$$

With:

$SP(t)_{c,n}$	is the total number of individuals that engage in sports in category of sports c (i.e. fitness or sports club) in neighborhood n at time t
$Fac(t)_{c,n}$	is the total number of existing sports facilities of category of sports c (i.e. fitness or sports club) in neighborhood n at time t.

S9 Model calibration

The calibration process was performed using a grid search in which parameter sets of all unknown parameters were sampled from a uniform distribution. In total, eight parameters were calibrated (See Table S2). The model was run for 50 years to make sure it reached equilibrium. Model outcomes in equilibrium were matched to the observed overall sports participation and sports participation by age group, sex, income, and category of sports. The goodness-of-fit was assessed by maximizing the log-likelihood (LL) assuming a normal distribution. Optimal parameter values and the range of parameter uncertainty (95% confidence interval) were derived through a polynomial regression model. The model was calibrated under three assumptions of initial tendency: A) Gamma(1.0, 0.5), B) Gamma(1.0, 1.0), and C) Gamma(1.0, 3.0) (See also S2). The model assuming a shape parameter of 0.5 provided the best overall fit.

Table S2. Calibrated parameters.

Parameters		Initial tendency A. Gamma(1.0, 0.5) [a] Value	Low	High	B. Gamma(1.0, 1.0) [a] Value	Low	High	C. Gamma(1.0, 3.0) [a] Value	Low	High
Mean frequency of sports per year		153.5	146.2	160.9	50.3	48.5	52.1	20.9	20.4	21.5
a_groupS	*18-35yrs*	1.0	-	-	1.0	-	-	1.0	-	-
	35-54yrs	0.157	0.139	0.175	0.200	0.182	0.217	0.284	0.264	0.305
	>55yrs	0.148	0.136	0.160	0.180	0.169	0.190	0.242	0.230	0.254
sexS	*Male*	1.0	-	-	1.0			1.0	-	-
	Female	0.659	0.608	0.710	0.808	0.754	0.862	0.773	0.732	0.813
incomeS	*High income*	1.0	-	-	1.0	-	-	1.0	-	-
	Middle income	0.471	0.428	0.514	0.587	0.545	0.629	0.640	0.605	0.675
	Low income	0.428	0.387	0.470	0.465	0.421	0.509	0.547	0.511	0.582
β distance decay	*Fitness*	0.029	0.028	0.031	0.022	0.020	0.025	0.013	0.011	0.015
	Sports club	0.027	0.025	0.028	0.018	0.015	0.020	0.010	0.006	0.013
LL		35.3			20.0			-82.7		

[a] Variation in initial tendency follows a Gamma distribution with mean 1.0 and equal shape and rate parameter.

Population-level effects of interventions are the result of the average of 80 simulation runs. Uncertainty intervals (95%) reflecting parameter uncertainty were constructed by discarding the two highest and lowest outcome values of 80 simulation runs. Figure S4 compares the observed data with the model outcomes in equilibrium (i.e. after 50 years). Results in the manuscript were all based on the best fitted model, i.e. assuming a Gamma(1.0, 0.5) for variation of initial tendency.

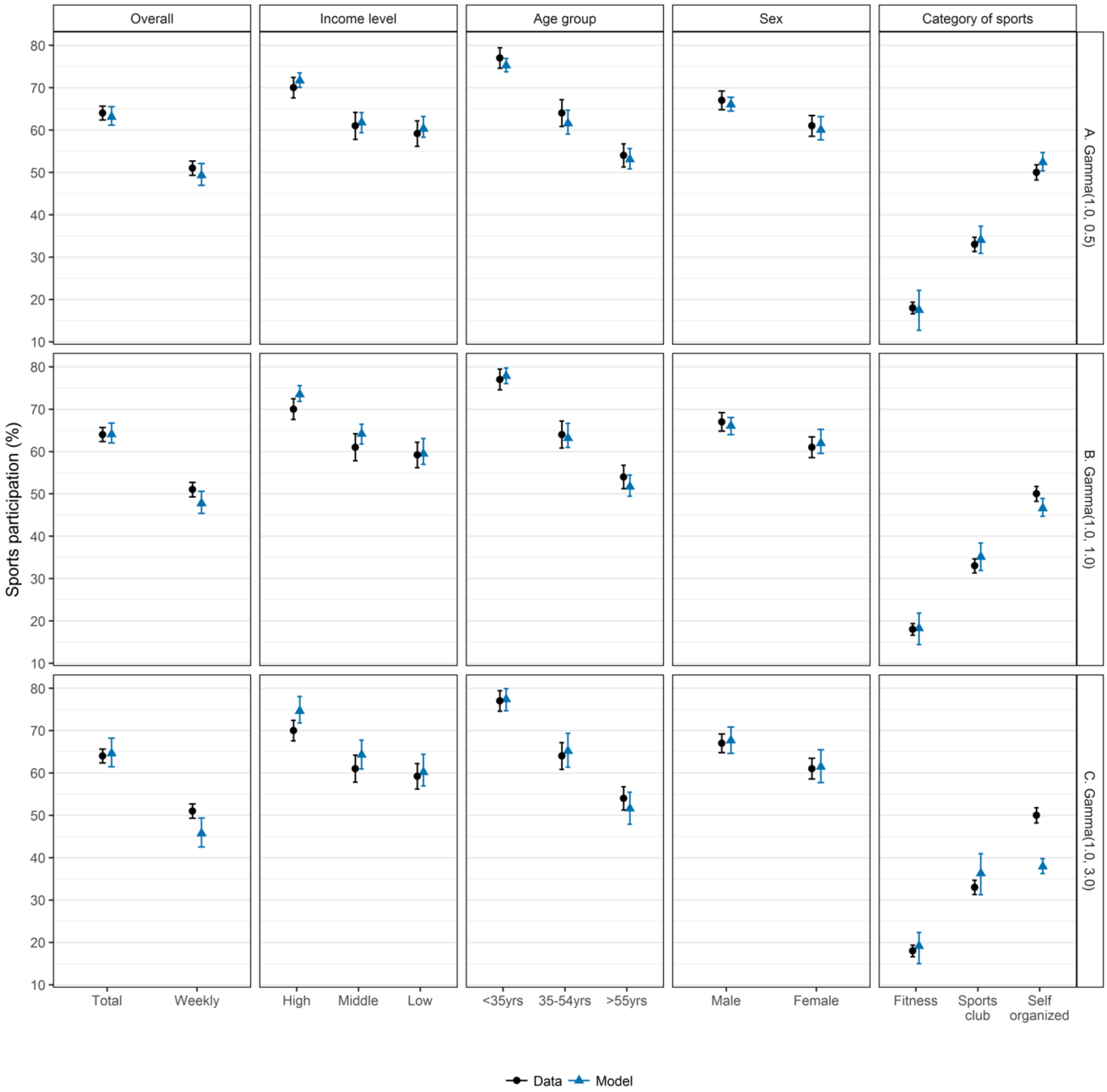

Figure S4. Comparison of modelled sports participation to the observed rates in Eindhoven in 2014. Model outcomes of overall sports participation, weekly sports participation, and overall sports participation by age group, sex, income level and category of sports (i.e. fitness, sports club, self-organized) are compared with data. Overall weekly sports participation included individuals who engage in 1) weekly sports in one of the categories of sports or 2) monthly sports at all three of the categories of sports (i.e. fitness, sports club, and self-organized). Model parameters were calibrated under three assumptions of initial tendency between individuals: A) Gamma(1.0, 0.5), B) Gamma(1.0, 1.0), C) Gamma(1.0, 3.0). Error bars represent the 95% uncertainty intervals reflecting parameter uncertainty and stochastic variation. Source data: Municipality of Eindhoven.

References

1. Statistics Netherlands. CBS Statline. 2016. https://opendata.cbs.nl/statline/#/CBS/nl/. Accessed March 2016.

2. Statistics Eindhoven. Eindhoven in cijfers. 2016. https://eindhoven.incijfers.nl/jive. Accessed Aug 2016

3. Municipality of Eindhoven. Einhoven sport. 2016. https://eindhovensport.nl/. Accessed Jan 2016.

4. Directorate-General for Education and Culture, *Special Eurobarometer 412 'Sport and physical activity'*. 2014, European Commission: Brussels.

5. Comensis. Fitness register. 2016. http://fitnessregister.nl. Accessed Jan 2016

6. Van Spronsen & Partners. Het fitnesscentrum in beeld. 2013. https://www.spronsen.com/brancheinfo/. Accessed Aug 2016

7. Simon HA, Rationality in Psychology and Economics. Journal of Business, 1986. **59**(4): p. S209-S224.

8. Hendriksen T, Hoogwerf I. Sportersmonitor 2012. 2013. Dongen: GfK.

9. Chamber of commerce. Brancheinformatie. 2016. www.ondernemersplein.nl/brancheinformatie. Accessed Jan 2016
